# New Appliances and Things Medical

**Published:** 1897-02-06

**Authors:** 


### NEW APPLIANCES AND THINGS MEDICAL.

**[We shall be glad to receive, at our Office, 28 & 29, Southampton Street, Strand, London, W.C., from the manufacturers, specimens of all new preparations and appliances which may be brought out from time to time.]**

### LEE'S PATENT AUTOMATIC DISINFECTOR.

(Auto-Disinfector Co., 193, Northumberland Street, Liverpool.)

This apparatus automatically supplies a given quantity of disinfectant every time a flush-out tank or cistern is emptied, and hence for lavatories and closets it is an economical means of disinfecting. The disinfector consists of a metal can, with a syphon outlet, which is protected by a fine brass wire gauze to prevent choking. When the cistern fills the can is also filled from the tap, or inlet, and the water coming in contact with the contents part is dissolved. When the cistern is emptied and the water sinks below the level of the outlet, by its syphon action the contents are poured out into the cistern and, mingling with the residual water, enter the pan, sink, or drain which it is intended to supply. In this way automatic economic disinfection is provided for. Nothing can go wrong, and no mechanism can get out of gear. The apparatus is simplicity itself. The difficulty is to find a satisfactory disinfectant for filling the can. It must not be too soluble, or else the disinfector will soon become emptied and necessitate refilling; it must be a powerful germicide and oxidiser or else it will be of no use. The can forwarded for our inspection was filled with permanganate of potash. This to our mind is a most unsatisfactory antiseptic for the purpose. To begin with it is too soluble, and, secondly, the staining which it causes to the pan (if used for closets) is such as to render it very objectionable for private houses, where they are usually of white glazed earthenware. But the idea of the automatic disinfector is excellent, and, with a suitable antiseptic, it would be a most desirable and hygienic addition to the sanitary arrangements of private houses as well as of public lavatories.

### VACCINE VIRUS.

(H. M. Alexander and Co., 35, Snow Hill, E.C.)

We have received a few tubes of the above virus, and find that they produce the typical vesication and symptoms without signs of extraneous contamination. The method that has been adopted by the firm for the preparation of the virus appears to be beyond reproach, both as regards ensuring efficacy of the vaccine and the avoidance of contamination with tuberculous and other pathogenic organisms. We can confidently recommend this vaccine to the notice of our readers.

### SPHAGNOL SOAP.

(Peat Products, Limited, 70, Lincoln's Inn Fields.)

Sphagnol is a new name given to a more or less new substance, which is obtained by the distillation of peat. It is a tarry product, stated to resemble in certain particulars ichthyol, which is itself a variety of earth oil. Its value as a therapeutic agent is believed to be due to a certain proportion of creasote and sulphur which it contains; its reputed efficacy in cases of acne, herpes, and eczema may be consequently explicable on the same grounds as have been adduced in the case of ichthyol. In the sample of soap, ordinary-sized tablet, which has been forwarded us for inspection and trial, it is claimed that 200 grains of sphagnol have been incorporated. The soap itself appears to be of good quality and free from caustic alkali, and if, as claimed for it, it possesses therapeutic properties allied to those of ichthyol, it is probable that it has a useful future before it, not only as a handy domestic application, but as a therapeutic agent of definite action in the hands of the physician.

### PEPTARNIS.

(Liebig's Extract of Meat Company, Limited, 9, Fenchurch Avenue, London E.C.)

This valuable preparation of beef, which heretofore has passed under the name of "Peptonum Carnis," or peptone of beef, is one of the most useful of the many concentrated invalid foods on the market. Various analyses show that it consists to the extent of nearly 60 per cent. of digested meat proteids and extractives, while the remaining 40 per cent. is made up of mineral constituents and water, results which, as far as concentration is concerned, are almost unrivalled in the manufacture of pre-digested meat foods. It is the large percentage of albumoses and peptones that renders Peptarnis so specially serviceable for invalid use, since these proximate principles are believed to be absorbed from the stomach and intestines direct without making any calls upon the natural supplies of physiological energy. The strength of the invalid is thus at once protected, and the stimulating and tonic effects of this condensed food are manifested throughout the system in a shorter space of time than is the case with almost any other nutritive substance. So many misconceptions have from time to time arisen as to the exact value of meat extracts in general and peptones and albumoses in particular, that it cannot be too clearly stated that as they exist in Peptarnis they are not only foods, but foods in a physiological sense of extreme economy.

